# Assessing groundwater dynamics in data-scarce mountainous regions using a lumped parameter groundwater model

**DOI:** 10.1007/s44195-025-00112-x

**Published:** 2025-08-26

**Authors:** Ya-Sin Yang, Hsin-Fu Yeh, Chia-Chi Huang, Jonathan D. Mackay, John P. Bloomfield, Kuo-Chin Hsu, Shien-Tsung Chen

**Affiliations:** 1 Department of Resources Engineering, National Cheng Kung University, Tainan 701, Taiwan (ROC); 2 British Geological Survey, Environmental Science Centre, Keyworth, Nottingham NG12 5GG, UK; 3 British Geological Survey, Maclean Building, Benson Lane, Crowmarsh Gifford, Wallingford, Oxfordshire, OX10 8BB, UK; 4 Department of Hydraulic and Ocean Engineering, National Cheng Kung University, Tainan 701, Taiwan (ROC)

**Keywords:** Groundwater level, Mountainous groundwater, Simulation, Lumped parameter model, AquiMod

## Abstract

Given the pressures on water resources caused by global climate change and human activities, the assessment and management of groundwater resources in mountainous region have become increasingly important. The central mountainous region of Taiwan, as one of the significant sources of groundwater recharge, plays a critical role in overall water resource management due to its groundwater storage capacity and recharge capability. Addressing the challenges of limited survey and observational data in mountainous groundwater assessments, this study uses the lumped parameter groundwater model AquiMod to analyze long-term groundwater level changes at 23 monitoring stations in mountainous areas of central Taiwan. This study is based on long-term groundwater level monitoring data (2010–2021) analyzing the relationship between groundwater levels and precipitation, and performs model calibration and prediction. The results indicate a strong correlation between groundwater levels in mountainous areas and precipitation. While the model predictions were satisfactory for most monitoring stations, obtaining Nash Sutcliffe efficiency scores of between 0.5 and 0.9 at 14 of the 23 monitoring stations. However, poorer performance at several stations reflects limitations arising from data gaps, complex local geology, and the inability of the lumped model to represent lateral recharge or anthropogenic influences. Model sensitivity analysis further highlights the critical role of unsaturated zone parameters, such as rooting depth, soil storage and upper-layer saturated hydraulic conductivity, in shaping groundwater responses. In summary, the lumped parameter groundwater model has proven practical for evaluating groundwater in Taiwan’s mountainous regions and can serve as a reference for the sustainable management of future water resources.

## Introduction

Mountainous groundwater is an important source of water for drinking, agriculture and industry for many regions and their downstream end users. It is essential for water resource protection and environmental conservation, and plays a significant role in disaster prevention, geological stability, and climate change adaptation (Taylor et al. 2013). Groundwater is crucial in mountainous hydrological systems, serving as a stable and significant water source of recharge for surface water. Its contribution is particularly pronounced during the dry season, when groundwater often dominates streamflow and sustain river runoff. Furthermore, groundwater possesses certain natural recovery capabilities (Somers et al. 2019), which are contingent upon precipitation and infiltration processes. However, it is also susceptible to long-term climate change impacts and variations in human activities across different environments and time scales (de Jong 2015). With the increasing demand for water, Viviroli et al. (2020) indicated that water resources in lowland areas will become increasingly dependent on mountainous regions. Persistent groundwater depletion could lead to severe water use risks. Under climate change, changes in precipitation patterns and increased evaporation could lead to a decline in groundwater reserves, subsequently reducing mountainous runoff and threatening water resources (Carroll et al. 2024). Therefore, protecting and managing mountainous groundwater resources is crucial for addressing climate change and ensuring the sustainability of water resources.

Extensive and continuous groundwater level data are essential for understanding and managing groundwater resources (Alley et al. 2002). Groundwater level is a direct and straightforward measure of groundwater availability and accessibility. Groundwater level encompasses a comprehensive response to various climate, topographical, and hydrogeological factors and their interactions, making the simulation of groundwater level a challenging task (Afzaal et al. 2019; Davamani et al. 2024). Multiple groundwater modelling approaches have been employed to simulate groundwater levels, among which physically based process-driven models continue to be the most widely used (Gogu et al. 2001; Ashraf and Ahmad 2008; Khan et al. 2017; Condon et al. 2021). These models simulate groundwater flow based on the physical laws of fluid dynamics, typically involving complex equations. Their advantage lies in the fact that the parameters are often related to known hydrogeological characteristics (Kresic and Mikszewski 2012; Anderson et al. 2015). Solving these equations usually involves numerical methods to approximate values over a multi-dimensional spatial grid and time, resulting in significant computational demands and the need for extensive data. As a result, the modeling and execution costs are high, with a strong sensitivity to data quality and model parameters (Ojha et al. 2015). Hence, a thorough understanding of the physical processes involved is crucial for selecting an appropriate model (Kirchner 2006; Menichini et al. 2022).

Another approach is the use of data-driven (empirical) models, which do not require prior knowledge of physical processes but instead rely on empirical relationships between groundwater levels and one or more predictor variables (Shirmohammadi et al. 2013; Wu et al. 2021; Sarma and Singh 2022; Tao et al. 2022). Common methods include time series analysis (Mirzavand and Ghazavi 2015; Dadhich et al. 2021; Zarinmehr et al. 2022), regression models (Sahoo and Jha 2013; Huang et al. 2019; Elbeltagi et al. 2022), and machine learning approaches (Kenda et al. 2018; Müller et al. 2021; Osman et al. 2022). Although these models are relatively easy to implement and computationally cost-effective, they often lack the capability to provide a mechanistic understanding of system behaviour. They are typically applied in situations where data is abundant and the physical processes are difficult to capture (Wei et al. 2020). Data-driven models require training prediction models using observed data and are often considered black-box models due to their lack of explicit physical processes and mechanisms. As a result, they are generally unsuitable for predicting groundwater levels in complex environments (Yin et al. 2021).

Lumped parameter groundwater models represent an alternative approach that retain some physical principles of groundwater systems while simplifying some of the complexities inherent in physically based models (Mackay et al. 2014). These models are typically represented by a conceptual framework that describes the hydrological system, composed of storage modules that represent key hydrological processes such as precipitation, infiltration, and groundwater flow. This framework allows for the configuration of different model structures (Birtles and Reeves 1977). Furthermore, these models can be evaluated and constrained based on field data, offering advantages of rapid operation and low cost. They require minimal specialized modeling knowledge and are suitable for situations with limited data availability (Ejaz et al. 2022). Their runtime efficiency also makes them ideal for application to large (e.g., national-scale) groundwater level monitoring datasets (Collenteur et al. 2023). Its simplicity and practicality make it highly valuable for preliminary assessments and management decisions. AquiMod is a lumped groundwater model developed by the British Geological Survey (Mackay et al. 2014, 2022). The model simulates groundwater level time series at boreholes in unconfined aquifers by integrating simple conceptual hydrological algorithms for soil drainage, water transport through the unsaturated zone, and groundwater flow. A key feature of AquiMod is its ability to represent multiple groundwater flow pathways and incorporate vertically heterogeneous hydraulic conductivity parameters. It has been demonstrated to effectively capture nonlinear groundwater level dynamics across a range of hydrogeological environments (Prudhomme et al. 2017). The model has been successfully applied to seasonal groundwater level forecasting (Mackay et al. 2015), groundwater level reconstruction (Jackson et al. 2016; Ascott et al. 2020), assessing the impact of climate change on groundwater levels (Ascott et al. 2022), and evaluating groundwater recharge rates (Seidenfaden et al. 2023).

Approximately 70% of Taiwan’s land area consists of mountainous and hilly terrain. With the ongoing social and economic development, the water resources in the plains have gradually become insufficient to meet the growing demand. Over-abstraction of groundwater has led to land subsidence issues in some plain areas (Hsu et al. 2015; Shih et al. 2019; Lu et al. 2020). The development of water resources in Taiwan is constrained by social pressures, with the construction of reservoirs and watershed facilities facing significant obstacles. As a result, groundwater resources have emerged as a potentially important alternative water source. The precipitation characteristic of Taiwan’s mountainous regions are significantly influenced by monsoon and orographic effects, with an average annual precipitation of approximately 2500 mm, considerably higher than the global average. Precipitation is unevenly distributed in both time and space, primarily concentrated during Mei-Yu season (May to June) and typhoon season (July to October). Orographic lifting often enhances rainfall with increasing elevation, leading to annual precipitation exceeding 3000 mm in some high-altitude areas (Chen and Chen 2003; Agyakwah and Lin 2021). These characteristics make Taiwan’s mountainous regions critical recharge zones for surface and groundwater resources, playing a vital role in water resource management. Mountainous areas serve as crucial recharge sources for Taiwan’s coastal plain aquifers, and the groundwater storage capacity and recharge ability of these regions are vital for overall water resource management (Yeh et al. 2009, 2014; Huang et al. 2013; Chen et al. 2022). Since 2010, the Geological Survey and Mining Management Agency, Taiwan has initiated a “Comprehensive Project on the Investigation and Research of Mountainous Groundwater Resources in Taiwan.” This project involves collecting hydrological and geological data at the watershed scale, constructing monitoring wells in mountainous areas to gather continuous groundwater level data, and developing a hydrological and geological database for these regions. These data represent a unique mountain groundwater monitoring dataset and a unique opportunity to evaluate the potential for using conceptual groundwater models for simulating groundwater level dynamics in mountain aquifers.

This study focuses on developing a groundwater level prediction model for the central mountain region of Taiwan to support the sustainable management of water resources. Due to the general lack of high-altitude hydrological and meteorological data at the watershed scale in mountainous regions, this study uses AquiMod to develop a point-scale groundwater level model for assessing groundwater in these areas. This study is guided by two core hypotheses. First, long-term trends in groundwater levels are significantly influenced by precipitation pattern, and analyzing these trends can provide insights into the sustainability of groundwater resources in mountainous regions. To test this, we analyzed long-term groundwater level data to identify trends and assessed the relationship between groundwater levels and precipitation. Second, a simple lumped conceptual groundwater can effectively simulate groundwater dynamics in mountain aquifers, despite the inherent geological and hydrological complexities. To evaluate the hypotheses, we employed the lumped conceptual groundwater model AquiMod to simulate and predict groundwater levels, assessing its performance under various geological and hydrological conditions. The findings of this study can contribute to future research and management of mountainous groundwater resources.

## Materials and methods

### Study area and groundwater level observations

#### Hydrogeological background

**Fig. 1:**
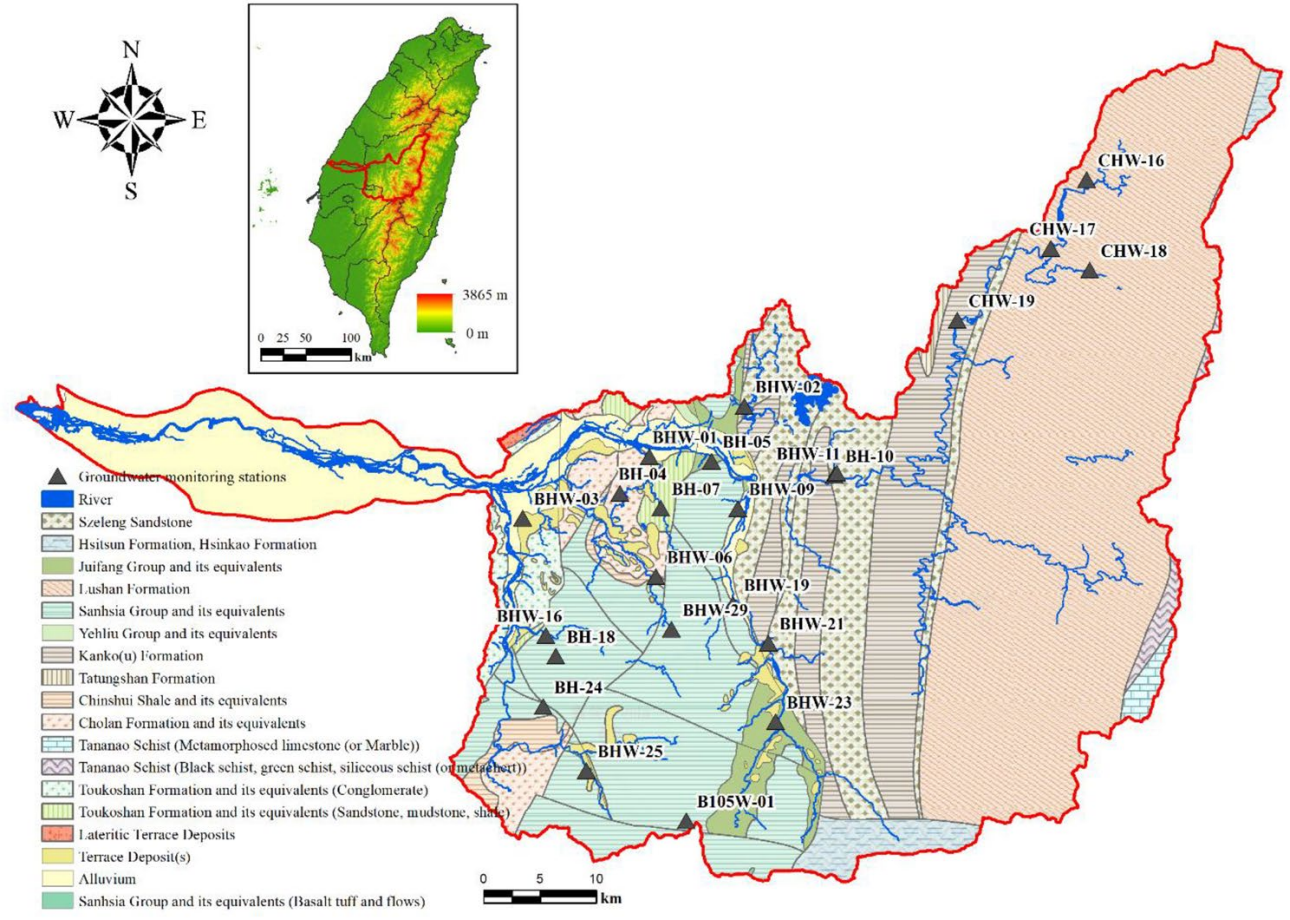
Geological map of the Choushui River Basin

The study area is located in the Choushui River Basin in the central mountain region of Taiwan, covering an area of approximately 3,156 square kilometers, making it the second-largest river basin in Taiwan (Fig. 1). The basin exhibits significant elevation differences. In the upstream region, the elevation changes from approximately 3,500 m above sea level to around 500 m, with an average river slope of about 0.03. In the midstream, the elevation decreases from 500 m to around 50 m, with an average river slope of about 0.01. In the downstream, the elevation changes from 50 m to approximately 0 m, with an average surface slope ranging from 0.001 to 0.003 (Water Resources Planning Branch 2003).

The Choushui River Basin’s geology varies across its upper, middle, and lower reaches, corresponding to three different geological regions of Taiwan, as shown in Fig. 1. The upper and middle reaches are situated in the western foothill geological region, where the strata are primarily composed of Tertiary and Quaternary sedimentary rocks. The main lithologies include sandstone, shale, sandy shale interlayers, and conglomerate layers. In the lower reaches, the geology consists primarily of unconsolidated modern alluvial deposits, including clay, silt, sand, and gravel, with the gravel mainly composed of sandstone or quartzite. These modern alluvial deposits are predominantly found in the riverbed and the alluvial fan areas along both banks, particularly in the lower reaches of the Choushui River. The geological formations of the basin, from west to east, include Miocene to Pliocene sandy shale layers, Eocene to Oligocene slate and quartzite layers, and Miocene shale and metamorphic sandstone layers. Due to the widespread distribution of slate and hard shale, which are prone to weathering and erosion, the Choushui River frequently carries significant amounts of silt and sediment, resulting in turbid river water (Kao and Milliman 2008; Kuo et al. 2017; Deng et al. 2020).

The aquifer systems in this region is generally shallow and unconfined, consisting of colluvial deposits that range in thickness from a few meters up to several tens of meters. These deposits typically overly fractured and less-permeable bedrock composed of slate, shale, or meta-sandstone, forming a complex hydrogeological setting characterized by variable hydraulic conductivity and heterogeneity(Chen et al. 2022).

Groundwater recharge primarily occurs via direct infiltration of precipitation, given the steep topography and the absence of significant irrigation or groundwater abstraction in the mountainous region. The area receives annual precipitation of over 2,000 mm, mostly from typhoons and the summer monsoon, contributing to rapid infiltration into the colluvial layer and downward percolation along fractures or faults. The recharge is strongly influenced by slope aspect, vegetation cover, and antecedent moisture conditions, leading to spatially variable recharge rates.

Previous groundwater studies in the Choushui River Basin have mainly focused on the alluvial plains or regional-scale water balance assessments(Yu and Chu 2010; Ke 2014; Yeh et al. 2022), with fewer investigations targeting the mountain regions(Chen et al. 2023). Chen et al. (2022) evaluated the response of shallow aquifers in hilly terrain and suggested the dominance of local flow systems and high recharge sensitivity to precipitation. This study focusses on the middle to upper reaches of the basin, where monitoring wells are located in shallow colluvial deposits and fractured bedrock. In this mountainous region with limited hydrogeological data, groundwater levels are expected to be primarily influenced by climatic inputs such as precipitation. This study uses groundwater level observations from wells in shallow colluvial deposits and fractured bedrock to explore the feasibility of simulating groundwater dynamics using a simplified conceptual model.

#### Groundwater monitoring well information

The study area includes 29 groundwater monitoring wells, as listed in Table 1, and the locations of these wells are indicated in Fig. 1. The data are sourced from the Taiwan Hydrological and Geological Information System. As of 2022, 6 wells are decommissioned, and 23 are actively in use. These wells are primarily designed as screened well, enabling the measurement of groundwater level within specific geological unit. The screening locations of the wells are specifically intended to monitor changes in groundwater levels within the colluvial layer and the fracture bedrock areas connected to this layer. The screening depth and filter pack depth for each well, along with their corresponding monitoring zone and lithology, are presented in Table 1. Among the wells currently under monitoring, 6 are located in bedrock (BH-10, BH-18, BH-24, BHW-19, BHW-21, CHW-16), while the remaining wells are situated in regolith. This study used data from the 23 wells with continuous monitoring for subsequent analysis. The groundwater level data used in this study were not interpolated or gap-filled; instead, the original observations were retained to preserve the authenticity of hydrological variations. No artificial pre-processing methods, such as imputation or smoothing, were applied. To ensure consistency across the 23 selected stations, uniform data selection criteria were adopted, and the observation periods were aligned accordingly. This approach emphasizes the use of actual measured data and reflects the inherent variability and limitations of field-based hydrological monitoring in mountainous regions.

**Table 1:** Status of groundwater monitoring stations in the study area

Station No.	TWD97_X (m)	TWD97_Y (m)	Elevation (masl)	Screened depth (m)	Filter pack depth (m)	Monitoring Zone	Lithology	Record period	Status
B105W-01	230708.6	2601451.5	2203.81	30 ~ 42	7 ~ 45	Regolith	Sandstone-dominated interbedded sandstone and shale	2016–2022	Active
BH-04	224828.0	2630459.0	406.27	15 ~ 27	11 ~ 43	Regolith	Sandstone	2010–2022	Active
BH-05	232953.0	2633304.0	286.88	27 ~ 39	6 ~ 43	Regolith	Colluvium; Accumulated debris; Debris	2010–2022	Active
BH-07	228464.0	2629142.0	327.69	12 ~ 20; 24 ~ 28	11 ~ 52	Regolith	Argillaceous sandstone	2010–2022	Active
BH-08	233519.0	2630322.0	835.02	20 ~ 32	6 ~ 36	Regolith	Sandstone	2010–2014	Inactive
BH-10	243875.0	2632065.0	375.46	27 ~ 39	8 ~ 43	Bedrock	Quartzite	2010–2022	Active
BH-18	219129.0	2616045.0	631.53	27 ~ 39	8 ~ 39	Bedrock	Sandstone	2010–2022	Active
BH-20	228331.0	2615107.0	1662.91	48 ~ 60	8 ~ 68	Bedrock	Sandstone	2010–2018	Inactive
BH-22	235382.0	2613460.0	934.10	16 ~ 24; 28 ~ 32; 36 ~ 40; 48 ~ 52	11 ~ 56	Bedrock	Sandstone	2010–2013	Inactive
BH-24	218025.0	2611566.0	752.45	22 ~ 34	8 ~ 37	Bedrock	Sandstone	2010–2022	Active
BH-26	242597.0	2606512.0	1141.16	13 ~ 25	12 ~ 29	Regolith	Siltstone; Interbedded sandstone and shale	2010–2021	Inactive
BH-27	221545.0	2629230.0	186.06	22 ~ 34	7 ~ 38	Regolith	Sandstone	2010–2014	Inactive
BHW-01	227444.0	2633698.0	218.29	33 ~ 45	7 ~ 48	Regolith	Argillaceous sandstone	2010–2022	Active
BHW-02	235877.0	2638191.0	455.27	6 ~ 18	5 ~ 24	Regolith	Shale	2010–2022	Active
BHW-03	216180.0	2628261.0	139.71	40 ~ 52	34 ~ 55	Regolith	Gravel	2010–2022	Active
BHW-06	228027.0	2623089.0	781.17	13 ~ 25	7 ~ 25	Regolith	Sandstone	2010–2022	Active
BHW-09	235284.0	2629120.0	388.61	37 ~ 49	7 ~ 52	Regolith	Colluvium; Accumulated debris; Debris	2010–2022	Active
BHW-11	244048.0	2632254.0	385.45	44 ~ 56	15 ~ 59	Regolith	Gravel	2010–2022	Active
BHW-16	218249.0	2617868.0	281.38	27 ~ 39	14 ~ 42	Regolith	Sandstone	2010–2022	Active
BHW-19	234849.0	2620592.0	510.55	24 ~ 36	18 ~ 39	Bedrock	Sandstone	2010–2022	Active
BHW-21	238016.0	2617172.0	632.62	41 ~ 53	17 ~ 56	Bedrock	Quartzite	2010–2022	Active
BHW-23	238638.0	2610213.0	769.66	9 ~ 21	6 ~ 24	Regolith	Sandstone	2010–2022	Active
BHW-25	221853.0	2605875.0	644.03	25 ~ 37	15 ~ 40	Regolith	Sandstone	2010–2022	Active
BHW-29	229407.0	2618409.0	1182.97	30 ~ 42	18 ~ 45	Regolith	Colluvium; Accumulated debris; Debris	2011–2022	Active
CH-20	258061.0	2649975.0	801.19	31 ~ 43	5 ~ 47	Bedrock	Sandstone	2011–2014	Inactive
CHW-16	266254.0	2658270.0	1240.41	60 ~ 72	6 ~ 75	Bedrock	Slate	2011–2022	Active
CHW-17	263060.0	2652166.0	930.66	13 ~ 25	5 ~ 28	Regolith	Slate	2011–2022	Active
CHW-18	266496.0	2650239.0	1170.75	33 ~ 45	5 ~ 48	Regolith	Slate	2011–2022	Active
CHW-19	254772.0	2645796.0	723.56	18 ~ 30	3 ~ 33	Regolith	Slate	2011–2022	Active

### Groundwater level trend analysis

#### Mann-Kendall trend test

The Mann-Kendall (MK) trend test (Mann 1945; Kendall 1975) is a non-parametric method used to identify and assess the significance of monotonic trends in a time series, without assuming normal distribution or linearity. Unlike parametric tests, the MK trend test does not require residuals to be normally distributed, making it robust for trend analysis (Hirsch and Slack 1984). It is effective in detecting significant upward or downward trends over time, even when data points are independent, not serially correlated, or contain missing values.

For a given time series {${x_i}$, *i =* 1, 2…, *n*}, the MK test assesses whether the data points are independently distributed (null hypothesis ${H_0}$) or if there is a monotonic trend (alternative hypothesis ${H_1}$). The test statistic S is calculated as follows:

1 $$S=\sum\limits_{i}^{{n - 1}} {\sum\limits_{{j=i+1}}^{n} {sign\left( {{x_j} - {x_i}} \right)} } $$

where n is the number of data points in the time series, and the $\:sign(\bullet\:)$ is the sign function, defined as:

2 $$sign({x_j} - {x_i})=\left\{ {\begin{array}{*{20}{c}} {1\begin{array}{*{20}{c}} {}&{\begin{array}{*{20}{c}} {if}&{{x_j} - {x_i}>0} \end{array}} \end{array}} \\ {0\begin{array}{*{20}{c}} {}&{\begin{array}{*{20}{c}} {if}&{{x_j} - {x_i}=0} \end{array}} \end{array}} \\ {\begin{array}{*{20}{c}} { - 1}&{\begin{array}{*{20}{c}} {if}&{{x_j} - {x_i}<0} \end{array}} \end{array}} \end{array}} \right.$$

when 𝑛 ≥ 8, the test statistic S approximately follows a normal distribution (Mann 1945; Kendall 1975). The mean $E\left( S \right)$ and variance $Var\left( S \right)$ of the statistic are given by the following:

3 $$E\left( S \right)=0$$

4 $$Var\left( S \right)=\frac{{n\left( {n - 1} \right)\left( {2n+5} \right) - \sum\limits_{{i=1}}^{m} {{t_i}\left( {{t_i} - 1} \right)\left( {2{t_i}+5} \right)} }}{{18}}$$

where *m* is the number of tied groups and ${t_i}$ represents the number in the *i*-th group. The standardized test statistic Z is calculated as follow:

5 $$Z=\left\{ {\begin{array}{*{20}{c}} {\frac{{S - 1}}{{\sqrt {Var(S)} }}\begin{array}{*{20}{c}} {}&{} \end{array}} \\ 0 \\ {\frac{{S+1}}{{\sqrt {Var(S)} }}} \end{array}} \right.\begin{array}{*{20}{c}} {}&{\begin{array}{*{20}{c}} {if}&{\left\{ {\begin{array}{*{20}{c}} {S>1} \\ {} \\ {S=0} \\ {} \\ {S<1} \end{array} } \right.} \end{array}} \end{array}$$

when $\left| Z \right|>{Z_{{\alpha \mathord{\left/ {\vphantom {\alpha 2}} \right. \kern-0pt} 2}}}$, it indicates a statistically significant trend in the time series. A positive 𝑍 value signifies an upward trend, while a negative 𝑍 value indicates a downward trend. Here, $\alpha$ represents the significance level, with $\alpha =0.05$ used in this study as the threshold for significance. Thus, if $\left| Z \right|>1.96$, it denotes a statistically significant upward or downward trend in the time series.

#### Theil-Sen estimator

The Theil-Sen estimator (Sen 1968, Theil 1950) is a robust statistical method for estimating the slope of a linear trend. It calculates the median of all possible slopes between pairs of data points, providing a resistant estimate to outliers and non-normal data distributions. Widely regarded as the most popular nonparametric technique for estimating linear trends, it often outperforms simple linear regression, even in normally distributed datasets.

The median slope is calculated as follows:

6 $$\begin{aligned}&\beta =median\left( {\begin{array}{*{20}{c}} {\frac{{{x_j} - {x_i}}}{{j - i}},}&{\begin{array}{*{20}{c}} {if}&{\begin{array}{*{20}{c}} {{x_i} \ne {x_j},}&{1 \leqslant i<j} \end{array}} \end{array}} \end{array} \leqslant n} \right)\cr&{j}=1,2,...,i+1...,i=1,2,...,n\end{aligned}$$

where $\beta$ is the median of all slopes between ${x_j}$and ${x_i}$ corresponding to the time j and i. When the slope $\beta$ is positive, it indicates an upward trend in the data; conversely, when the slope $\beta$ is negative, it indicates a downward trend.

### Cross correlation analysis between rainfall and groundwater level

The cross-correlation between two time series is a statistical measure used to assess the correlation between one time series and another at different time lags (Box et al. 2015). Specifically, cross-correlation quantifies the linear relationship between two time series as one is shifted in time relative to the other. In this study, the continuous groundwater level data is first processed through differencing to remove daily trends from the time series. Subsequently, the differenced groundwater levels are analyzed for correlation with precipitation data. The lagged cross-correlation function can be expressed as:

7 $$Cor{r_{XY}}\left( \tau \right)=\frac{{\sum\nolimits_{{t=1}}^{{T - \tau }} {\left( {{X_{t+\tau }} - \overline {X} } \right)\left( {{Y_{t+\tau }} - \overline {Y} } \right)} }}{{\sqrt {\sum\nolimits_{{t=1}}^{{T - \tau }} {{{\left( {{X_{t+\tau }} - \overline {X} } \right)}^2}\sum\nolimits_{{t=1}}^{{T - \tau }} {{{\left( {{Y_{t+\tau }} - \overline {Y} } \right)}^2}} } } }}$$

where $\tau$ represents the time lag, T is the total length of the time series, $\overline {X}$ and $\overline {Y}$ represent the mean values of X and Y, where X denotes precipitation and Y denotes the differenced groundwater levels (${G_{diff}}\left( t \right)=G\left( t \right) - G\left( {t - 1} \right)$) in this study. $Cor{r_{XY}}\left( \tau \right)$ value ranges from − 1 to 1, with values closer to 1 or − 1 indicating a stronger correlation of the sequence at lag $\tau$.

### Groundwater modeling

#### Groundwater model development

**Fig. 2:**
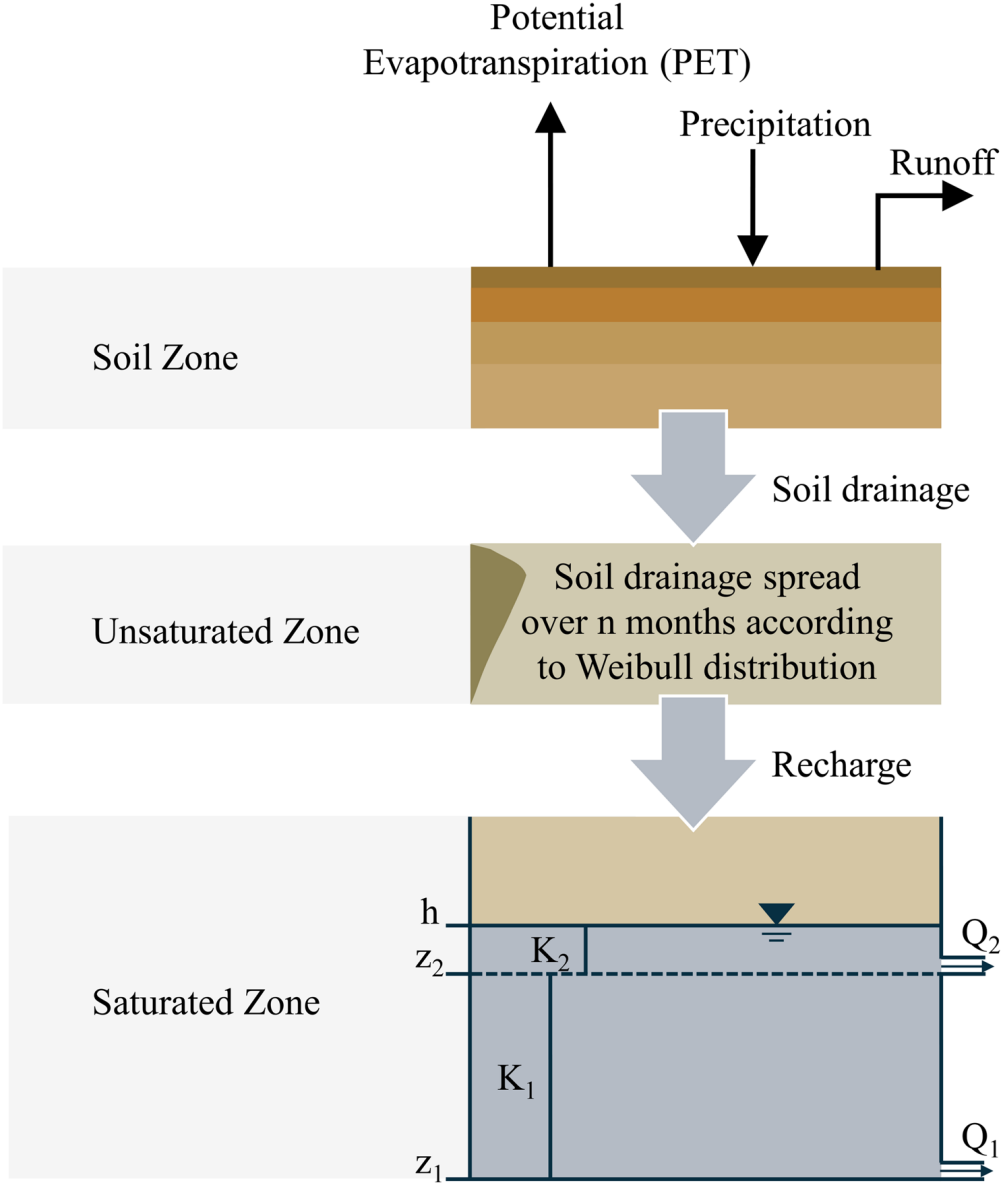
Conceptual framework of AquiMod. (Mackay et al. 2014)

In this study, the lumped groundwater model AquiMod was used. AquiMod is specifically designed for modeling groundwater level time series at observation boreholes (Mackay et al. 2014). As illustrated in Fig. 2, the conceptual framework of AquiMod consists of three modules: soil drainage, unsaturated zone water transport, and saturated groundwater flow. Soil drainage is estimated using the FAO method with a simplified soil water balance approach(Allen et al. 1998), followed by the assessment of the unsaturated zone using the Weibull distribution function. Saturated zone flow is calculated based on Darcy’s law, with the ability to represent variations in hydraulic conductivity with depth using up to three layers. The model requires time series of precipitation and potential evapotranspiration (PET) as driving data, along with observed groundwater level time series for calibration. For a complete description of the AquiMod, please refer to Mackay et al. (2014).

In term of aquifer conceptualization, AquiMod supports two main representations: (1) an unconfined aquifer mode, which simulates dynamic changes in groundwater level and saturated thickness, allowing for vertical heterogeneity; and (2) a simplified confined aquifer mode with fixed transmissivity, simulating only hydraulic head changes without considering multi-layer confined flow or variable storage properties.

This study focuses on groundwater level dynamics in the mountainous region of central Taiwan. Given that most monitoring wells are install on the regolith or unconfined systems, and considering the limited resolution and completeness of available hydrogeological data, this study adopted the unconfined aquifer conceptualization. For the saturated zone simulation, this study implemented a two-layer configuration was chosen based on the regional geological setting and available data resolution, which is sufficient to capture the main features of groundwater dynamics in the study area.

Additionally, while AquiMod can support multiple flow pathway structures and simplified flow direction modelling, the available data in this study were limited to time series observations, precluding the detailed representation of subsurface flow networks. Therefore, rainfall was treated as the primary recharge source, and processes such as groundwater pumping or additional recharge mechanisms were not included. These simplifications improve the model’s applicability under data-scared condition but also represent key limitation of this study.

#### Model calibration

Due to the sparse distribution of meteorological stations in mountainous areas and the lack of long-term, stable climate data, this study adopts gridded climate observation data provided by the Taiwan Climate Change Projection Information and Adaptation Knowledge Platform (TCCIP). The TCCIP data integrates various station data through statistical modeling, data imputation, and interpolation to provide long-term, continuous, high-resolution climate information, as summarized in Table 2. The TCCIP precipitation data, with a spatial resolution of 0.01 degrees of latitude and longitude, were derived from a total of 2,247 stations provided by various agencies, including the Central Weather Bureau, Environmental Protection Administration, Water Resources Agency, Forest Research Institute, Civil Aeronautics Administration, and Taiwan Power Company. The daily observational data, encompassing both active and decommissioned stations, were interpolated to generate a high-resolution gridded dataset from 1960 to 2022.

**Table 2:** Data information

Dataset	Time scale	Resolution	Period	Data variables
Gridded observation data	Daily	0.01°	1960 ~ 2022	• Precipitation
Taiwan historical climate reconstruction data	Daily	2 km	1980 ~ 2021	• Average wind speed • Relative humidity • Average temperature • Maximum temperature • Minimum temperature • Surface air pressure
TReAD solar radiation bias correction	Daily	0.01	1979 ~ 2021	• Solar radiation
Mountain groundwater in Choushui River Basin	Daily	Station	2010 ~ 2022	• Groundwater

Evapotranspiration data were calculated using the FAO-56 method (Allen et al. 1998) with a spatial resolution of 0.02 degrees of latitude and longitude. Climate data were derived from the TReAD (Taiwan ReAnalysis Downscaling Data) dataset, created by dynamically downscaling ERA5 reanalysis data with the Weather Research and Forecasting (WRF) model. TReAD provides high resolution climate data for Taiwan, covering the period from 1980 to 2021 and addressing limitations due to uneven station distribution. For additional details on TReAD’s methodology, variable and correction techniques, refer to Taiwan Climate Change Projection Information and Adaptation Knowledge Platform (2023).

The model calibration parameter ranges in this study are detailed in Table 3. The catchment length is defined as the distance from the monitoring station to the nearest downstream river. Due to the lack of site-specific data for unsaturated soil properties, default ranged were applied. The range of saturated hydraulic conductivity (K) was set to encompass the results of hydraulic tests. To ensure smooth model calibration, we extended the upper limit and assumed that the K values in the upper layer were higher than those in the lower layer. The top outlet elevation Z_2_ is positioned within the range of groundwater level fluctuations, while the bottom outlet elevation Z_1_ is set 5–20 m below the lower boundary of Z_1_. Calibration was performed using the SCE-UA global optimization algorithm (Duan et al. 1993), which is recognized for its robustness and efficiency in hydrological modeling (Yapo et al. 1998; Muttil and Jayawardena 2008; Huang et al. 2018). This study adopts the Nash-Sutcliffe Efficiency (NSE) scores to account for uncertainties in model structure and parameter selection. Only models having NSE scores exceeding 0.5 being considered acceptable.

**Table 3:** List of aquimod model parameters and calibration ranges

Station No.	Module												
	Soil						Unsaturated zone		Saturated zone				
	Δx (m)	BFI (–)	FC (–)	WP (–)	Zr (mm)	p (–)	k (–)	λ (–)	K_1_ (m d^–1^)	K_2_ (m d^–1^)	S (–)	Z_1_ (MASL)	Z_2_ (MASL)
B105W-01	1459	0.1–0.9	0.1–0.3	0.3–0.8	100–3000	0.1–0.9	1–7	1–5	0.001–100	0.1–500	0.001–0.1	2150–2160	2160–2192
BH-04	407											370–385	385–400
BH-05	6											160–270	270–280
BH-07	76											315–318	318–324
BH-10	130											350–360	360–370
BH-18	1624											580–595	595–618
BH-24	3141											735–740	740–745
BHW-01	60											210–212	212–214
BHW-02	15											440–448	448–452
BHW-03	1088											130–132	132–136
BHW-06	540											760–769	769–778
BHW-09	640											345–355	355–372
BHW-11	400											360–365	365–375
BHW-16	88											260–265	265–271
BHW-19	180											480–487	487–491
BHW-21	160											586–590	590–598
BHW-23	150											755–760	760–768
BHW-25	250											620–625	625–630
BHW-29	230											1150–1162	1162–1175
CHW-16	400											1175–1182	1182–1192
CHW-17	180											910–919	919–927
CHW-18	190											1130–1150	1150–1158
CHW-19	70											710–717	717–720

## Result and discussion

### Trend analysis of groundwater levels and precipitation influence

The trend analysis aims to test the first hypothesis, whether long-term trends in groundwater levels are significantly influenced by precipitation patterns. By examining the relationships between precipitation and groundwater levels over time, this study aims to determine the extent to which precipitation drives groundwater dynamics in this region. Table 4 presents the results of groundwater level trend analysis and its interaction with precipitation. Detailed correlation analysis between precipitation and groundwater levels at each station is provided in Appendix A. Significant increasing trends in groundwater levels were observed at BH-24, BHW-09, and CHW-19, while BHW-06 showed no significant trend. All other stations exhibited significant decreasing trends. To further explore the influence of precipitation, this study conducted a cross-correlation analysis between daily precipitation and groundwater levels. The results show that cross-correlation coefficients are highest and significant at lags of − 1 or 0 for most stations, suggesting that precipitation on the previous day and the current day are the main factors influencing daily groundwater level variations. Only CHW-16 showed a lag of 2 days. The daily groundwater data analysis indicates a strong correlation between precipitation and groundwater levels at the mountainous stations. These findings suggest that while some stations demonstrate positive groundwater responses to precipitation, the majority exhibits declining trends.

**Table 4:** Results of groundwater trend analysis and cross-correlation with precipitation

Station	Period	Daily GWL_mean_ (masl)	Fluctuation (m)	Z	β	Lag Time (days)	Corr_max_
B105W-01	2016–2021	2181.29	32.61	–9.48^***^	–0.00157	0	0.265
BH-04	2010–2021	384.55	26.41	–40.76^***^	–0.00289	0	0.531
BH-05	2010–2021	273.79	16.85	–34.03^***^	–0.00363	–1	0.332
BH-07	2010–2021	321.67	6.29	–2.47^*^	–0.00005	–1	0.405
BH-10	2010–2021	364.92	10.95	–4.65^***^	–0.00016	–1	0.465
BH-18	2010–2021	603.00	23.77	–10.60^***^	–0.00060	0	0.471
BH-24	2010–2021	741.95	5.24	13.91^***^	0.00015	0	0.643
BHW-01	2010–2021	212.48	3.33	–35.75^***^	–0.00028	0	0.327
BHW-02	2010–2021	449.00	5.16	–6.10^***^	–0.00007	0	0.531
BHW-03	2010–2021	134.04	4.12	–10.33^***^	–0.00008	–1	0.767
BHW-06	2010–2021	771.76	9.95	1.53^ns^	0.00004	0	0.428
BHW-09	2010–2021	356.32	20.07	10.78^***^	0.00033	–1	0.544
BHW-11	2010–2021	368.76	9.13	–30.21^***^	–0.00057	–1	0.707
BHW-16	2010–2021	264.39	7.69	–9.31^***^	–0.00006	–1	0.563
BHW-19	2010–2021	486.75	6.77	–5.07^***^	–0.00002	–1	0.611
BHW-21	2010–2021	590.45	10.07	–54.03^***^	–0.00064	–1	0.711
BHW-23	2010–2021	761.53	8.44	–6.78^***^	–0.00006	–1	0.672
BHW-25	2010–2021	627.92	4.58	–14.38^***^	–0.00024	–1	0.540
BHW-29	2011–2021	1164.73	14.42	–2.41^*^	–0.00006	–1	0.632
CHW-16	2011–2021	1185.32	10.91	–10.75^***^	–0.00025	–2	0.562
CHW-17	2011–2021	921.58	8.68	–15.84^***^	–0.00033	–1	0.545
CHW-18	2011–2021	1153.05	22.93	–24.77^***^	–0.00087	–1	0.178
CHW-19	2011–2021	717.25	5.39	4.30^***^	0.00005	–1	0.421

Fluctuation: GWL_max_ - GWL_min_. Significance level $\alpha =0.05$. *p* < 0.05 is denoted by *, *p* < 0.01 by **, *p* < 0.001 by ***; and *p* > 0.05 by ns (not significant)

The observed decreasing trends at most stations may be attributed to long-term variations in precipitation, seasonal drought, or sustained groundwater abstraction, which can gradually lower groundwater storage over time. Additionally, land use changes such as reduced infiltration capacity or increased impervious surfaces may also contribute to declining trends. In contrast, the increasing trends observed at BH-24, BHW-09, and CHW-19 could be related to localized recharge conditions, reduced pumping activities or specific hydrogeological settings that promote groundwater accumulation in these areas. These factors together suggest that the groundwater dynamics in the study region are influenced by both climatic variability and anthropogenic impacts.

This trend analysis also serves to identify long-term non-stationarity in groundwater levels, which can bias correlation results if not considered. By first assessing these trends, this study ensures that the correlation analysis isolates the short-term groundwater responses to precipitation from any underlying long-term declines or increases.

### Groundwater model calibration and validation

This study employed a 3-year spin-up period to ensure the model reaches dynamic balance before evaluation (Mackay et al. 2022). Daily observed precipitation and PET data from the three years preceding the simulation start date were used as driving data. The available groundwater level time series was divided into two subsets: 70% of the data was used for model calibration, and the remaining 30% for independent validation to verify model performance. Prediction was conducted using a set of optimal parameters for AquiMod that met the threshold of NSE > 0.5. Since solar radiation deviation data are available only up to 2021, the model evaluation in this study was conducted up to the end of 2021. The model performance metrics, including the Nah-Sutcliffe Efficiency (NSE) scores and normalized indictors, are summarized in Table 5. These metrics provide a quantitative assessment of the model’s fit to observed groundwater levels. These metrics encompass NSE, Normalized Amplitude Error (NAE), and Normalized Percentile Error (the normalized 5th and 95th percentile errors), providing a concise evaluation of the model’s capability to capture both amplitude and extremes of groundwater level fluctuations. The formula of NAE and percentile errors are presented below:

**Table 5:** Summary of NSE score and normalized metrics

Station No	Calibration				Validation			
	NSE	NAE	Normalized_5th_error	Normalized_95th_error	NSE	NAE	Normalized_5th_error	Normalized_95th_error
B105W-01	0.91	0.41	0.006	0.104	0.91	0.33	0.072	0.054
BH-04	0.37	0.30	0.101	0.046	-0.04	0.32	0.218	0.056
BH-05	0.46	0.41	0.209	0.042	-4.12	0.52	0.689	0.239
BH-07	0.70	0.36	0.038	0.026	0.80	0.13	0.077	0.027
BH-10	0.85	0.16	0.007	0.026	0.82	0.06	0.004	0.073
BH-18	0.85	0.38	0.096	0.014	0.84	0.15	0.126	0.057
BH-24	0.19	0.67	0.098	0.197	0.16	0.51	0.009	0.048
BHW-01	0.34	0.36	0.125	0.087	-1.34	0.30	0.285	0.007
BHW-02	0.59	0.28	0.053	0.018	0.61	0.37	0.194	0.026
BHW-03	0.89	0.09	0.023	0.034	0.92	0.04	0.109	0.016
BHW-06	0.84	0.23	0.002	0.094	0.85	0.08	0.035	0.167
BHW-09	0.73	0.13	0.021	0.023	0.82	0.23	0.012	0.008
BHW-11	0.85	0.15	0.043	0.010	0.20	0.17	0.180	0.160
BHW-16	0.84	0.21	0.021	0.020	0.79	0.21	0.049	0.017
BHW-19	0.90	0.08	0.019	0.029	0.76	0.22	0.015	0.046
BHW-21	0.57	0.18	0.050	0.032	-1.09	0.25	0.188	0.212
BHW-23	0.94	0.13	0.010	0.015	0.91	0.13	0.019	0.008
BHW-25	0.73	0.12	0.079	0.050	0.85	0.06	0.029	0.005
BHW-29	0.86	0.25	0.010	0.057	0.88	0.25	0.018	0.006
CHW-16	0.83	0.35	0.018	0.045	0.87	0.11	0.019	0.041
CHW-17	0.82	0.20	0.011	0.027	0.88	0.10	0.076	0.023
CHW-18	0.28	0.66	0.384	0.025	0.24	0.47	0.453	0.050
CHW-19	0.66	0.12	0.062	0.176	0.40	0.13	0.051	0.242

8 $$\begin{aligned}& {{\text{Normailized}}}\:{{\text{Percentile}}}\:{{\text{Error}}} \cr&\quad=\frac{{\left| { {{\text{Observed}}}\:{{\text{Percentile}}} - {{\text{Modeled}}}\:{{\text{Percentile}}}} \right|}}{{{{\text{Observed}}}\:{{\text{Max}}} - {{\text{Observed}}}\:{{\text{Min}}}}}\end{aligned}$$

9 $$\begin{aligned}& {{\text{Normailized}}}\:{{\text{Amplitude}}}\:{{\text{Error}}} \cr&\quad=\frac{{\left| { {{\text{Observed}}}\:{{\text{Amplitude}} - } {{\text{Modeled}}}\:{{\text{Amplitude}}} } \right|}}{{{{\text{Observed}}}\:{{\text{Amplitude}}} }}\end{aligned}$$

**Fig. 3:**
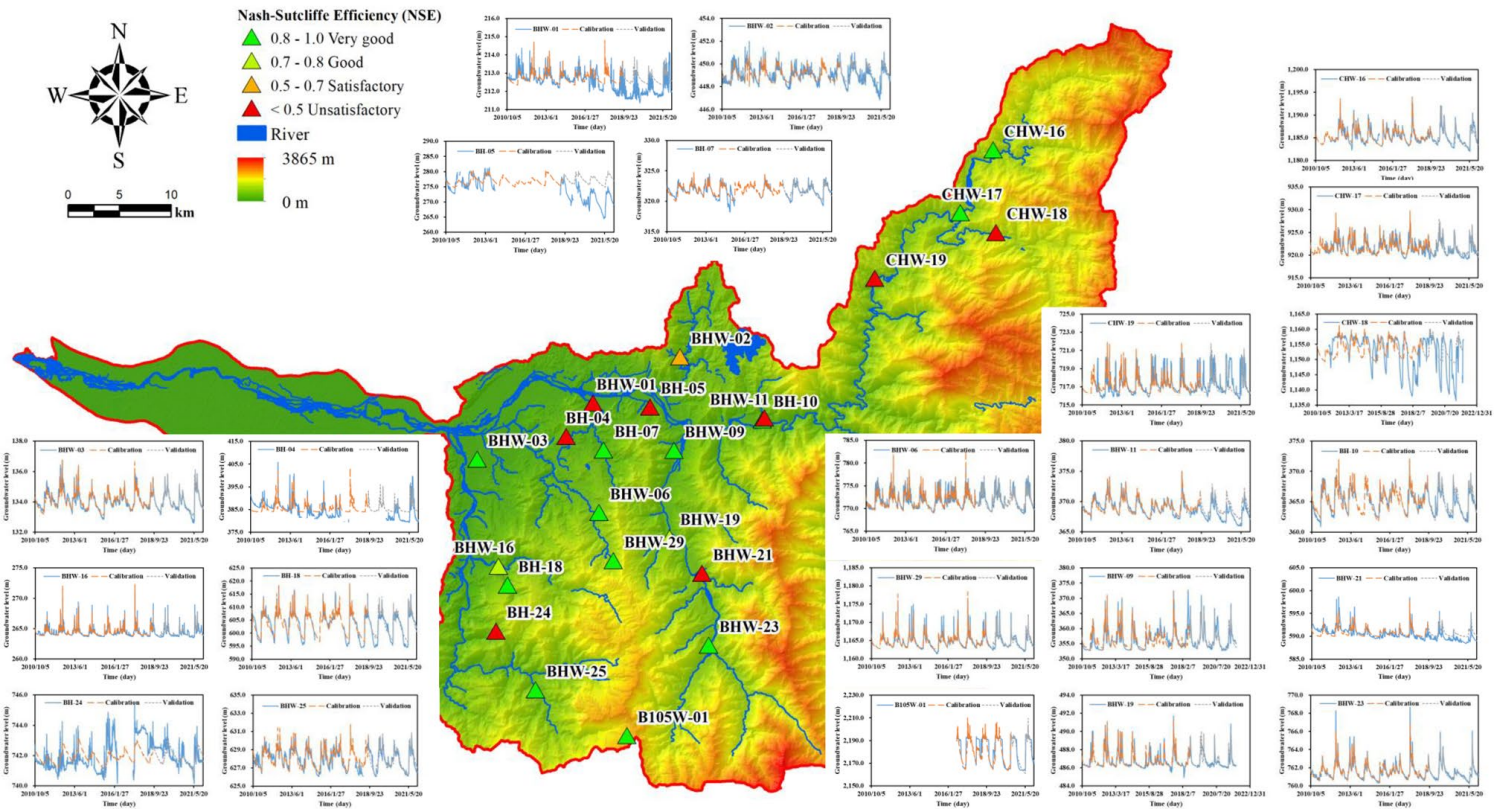
Best model’s NSE for monitoring wells in the study area

**Fig. 4:**
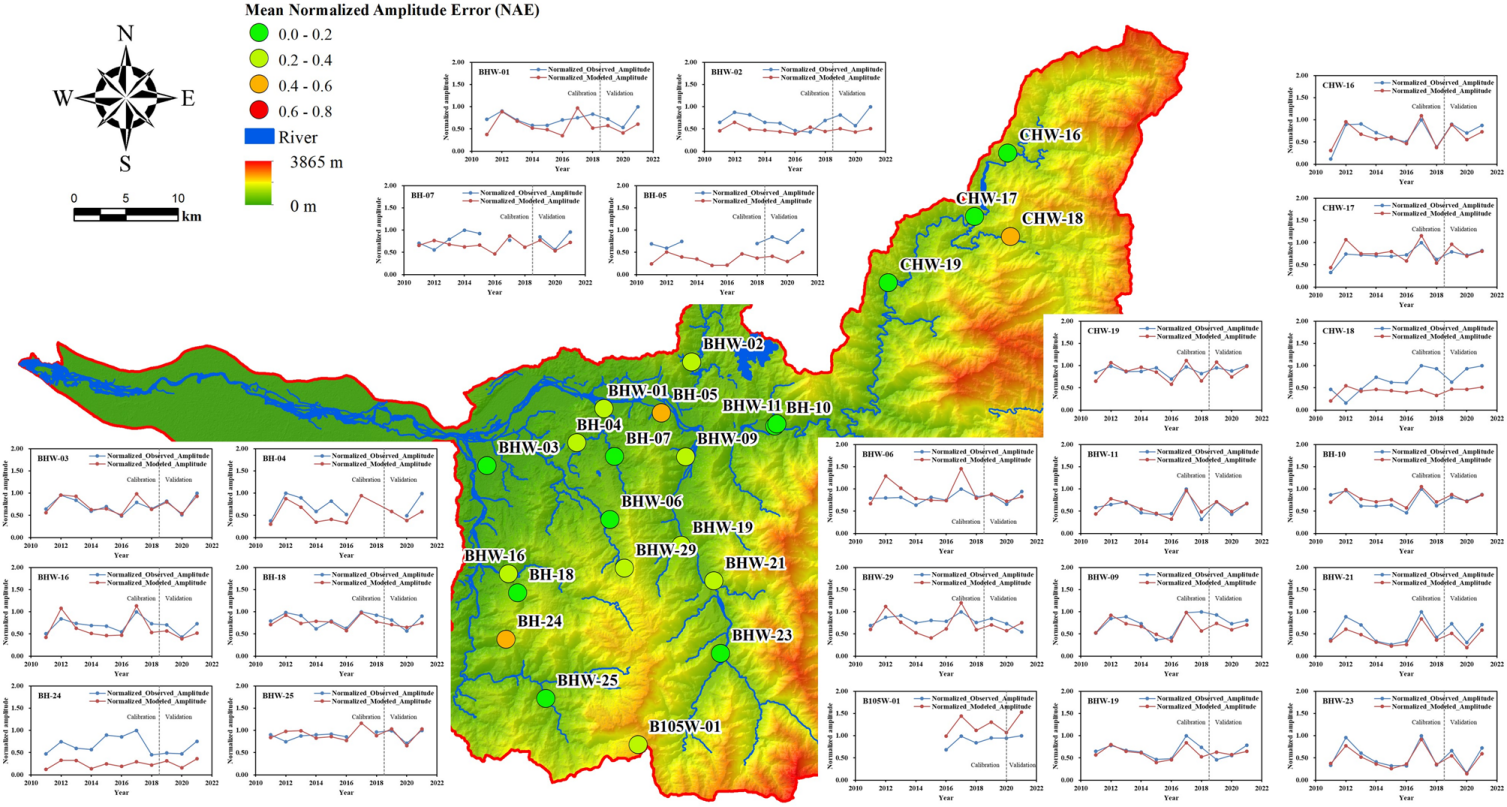
Normalized groundwater level fluctuation amplitude and mean NAE

The analysis of the groundwater model’s performance across 23 monitoring wells is summarized in Table 5. While the Nash-Sutcliffe Efficiency (NSE) how well the model reproduces temporal variations in groundwater levels, the Normalized Amplitude Error (NAE) reflects its accuracy in capturing the magnitude of fluctuations. Figures 3 and 4 illustrate the spatial distribution of NSE and NAE values, respectively, based on the model’s performance during the validation period. These figures highlight the spatial variability in model accuracy across the 23 monitoring wells, with NSE indicating temporal fit and NAE representing fluctuation magnitude errors.

During the calibration period, 15 out of 23 monitoring wells exhibited NSE values greater than 0.7, indicating good to very good model performance in capturing the temporal dynamics of groundwater levels. In contrast, poor performance was observed at wells such as BH-04, BH-05, BHW-01, BH-24 and CHW-18, where NSE values were below 0.5, suggesting that local hydrological processes or data limitations may not have been adequately represented. During the validation period, model performance remained relatively stable, with 14 wells maintaining NSE values above 0.7. However, several wells experienced marked declines in predictive accuracy, most notably BH-05 (NSE = -4.12), BHW-01 (NSE = -1.34), BHW-21 (NSE = -1.09), and BH-04 (NSE = -0.04). These negative NSE values may indicate model overfitting during calibration or temporal changes in hydrologic conditions, such as pumping activities variability, that were not accounted for in the model setup.

Closer inspection suggests that the negative validation performance at BH-04 and BH-05 may be attributed to data gaps during the calibration period, which limited the model’s ability to capture representative system behaviour. In contrast, BHW-01 and BHW-21 showed distinct downward trends in groundwater levels, possibly driven by long-term abstraction or seasonal deficits, which may not have been properly accounted for in the model structure. These issues likely led to suboptimal calibration and thus contributed to poor validation performance, highlighting the model’s sensitivity to data completeness and trend stability.

The spatial distribution of NAE values during the validation period reveals that most wells exhibit relatively low NAE, typically below 0.4, indicating that the model can reasonably capture the amplitudes. Meanwhile, the Normalized Percentile Error—representing deviations at the 5th and 95th percentiles of groundwater levels—provides insight into the model’s capability to reproduce extreme low and high water levels. As illustrated in Table 5, most wells showed NPE values within ± 20%, suggesting a satisfactory representation of both dry and wet period conditions. However, a few stations, particularly those with low observed amplitudes or sharp fluctuations, exhibited higher percentile errors. This discrepancy may result from local-scale influences not captured by the lumped model.

The performance of the groundwater model at well CHW-18 further highlights the challenges in simulating groundwater fluctuations in certain complex hydrogeological conditions. Located in the slate region on the western flank of Central Mountain Range, CHW-18 is situated in an area where the regional geology is characterized by well-developed cleavage. The strata have been folded due to gravitational forces along the cleavage planes, leading to tensile fractures and the formation of potential fault zones. These geological features contribute to the pronounced groundwater level fluctuations observed at CHW-18, which are significantly more extreme than those at nearby monitoring wells. The relatively low NSE score of 0.29 at CHW-18 suggests that the model struggles to accurately replicate the temporal trends of groundwater levels in this region. This is further evidenced by the relatively high NAE value of 0.57, indicating that the model also faces challenges in capturing the amplitude of groundwater level fluctuations. The presence of noticeable data gaps and trend changes in the groundwater hydrograph for CHW-18 may also contribute to the model’s difficulty in representing groundwater behaviour at this location. These factors, coupled with the geological complexity of the site, underscore the need for further refinement of the model to better account for local hydrogeological conditions at CHW-18.

Additionally, for some wells, the calibrated model fails to capture the full range of observed groundwater level extremes, including both maximum and minimum values. This discrepancy may reflect uncertainties in the observed groundwater levels, such as measurement errors, temporal resolution in monitoring, or localized disturbances that are not accounted for in the model. For instance, incomplete or low-quality observation data could result in an inaccurate representation of groundwater level fluctuations. Furthermore, these wells may exhibit distinct characteristics in terms of groundwater recharge. Such characteristics could include variations in recharge sources (e.g., direct precipitation versus lateral inflow), differences in aquifer properties like permeability and storage capacity, or the presence of preferential flow paths such as fracture or faults. These factors can significantly influence the timing and magnitude or recharge events, leading to discrepancies between observed and simulated extremes.

Due to practical considerations in the placement of monitoring wells, these groundwater monitoring stations are typically located in accessible, flat areas near rivers in the mountain valley. These locations may be influenced by surface-groundwater interactions and lateral recharge (Markovich et al. 2019). Although Taiwan’s mountainous regions experience less human activity compared to the plains, there is still local groundwater abstraction to meet the water demands of settlements and the tourism industry. Most wells in Taiwan, except for public and certain water rights wells, lack water meters to record abstraction volumes, making it difficult to accurately assess groundwater usage. According to data from the Water Resources Agency in Taiwan, the average annual registered groundwater abstraction in the study area is approximately 198.36 million cubic meters per year, equivalent to a daily abstraction rate of around 0.132 mm/day. In comparison, the average annual rainfall in the study area is 2,007 mm, equivalent to a daily average of 5.5 mm/day. Although rainfall significantly exceeds groundwater abstraction on a basin-wide scale, the concentrated groundwater abstraction in populated and flat area, particularly near river channels, may lead to localized groundwater depletion. Therefore, abstraction rates may surpass the natural recharge from precipitation, causing a decline in groundwater levels. Therefore, despite the overall abundant rainfall in this region, careful management of groundwater resources is essential to prevent potentially negative impacts such as localized over-extraction and the resultant decrease in groundwater levels.

### Sensitivity analysis of model parameters

**Fig. 5:**
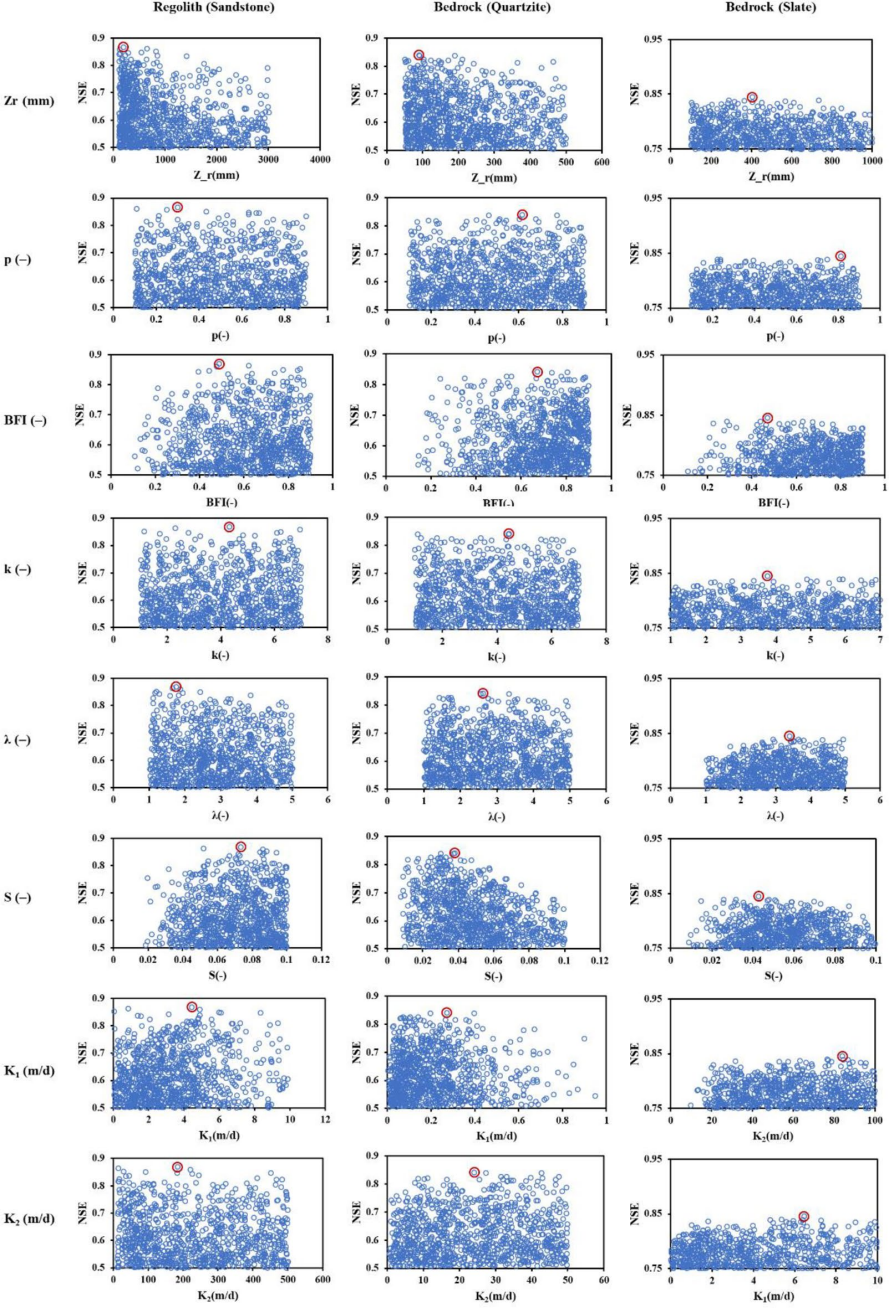
Dotty plots illustrating the sensitivity of each parameter. Blue dots represent indi-vidual simulation results obtained through Monte Carlo sampling, while the red dot indicates the parameter set yielding the highest model efficiency

A sensitivity analysis was conducted to evaluate the influence of individual model parameters on simulation performance across different geological settings. The analysis was based on a Monte Carlo framework in which 100,000 parameter sets were sampled uniformly within predefined ranges and evaluated using a lumped conceptual groundwater model. To identify behavioural parameter sets, an objective function threshold of 0.5 for the Nash–Sutcliffe Efficiency (NSE) was applied, and the number of acceptable models was limited to the top 1000 simulations that exceeded this threshold. The resulting dotty plots illustrate the relationship between each parameter and model performance, allowing the identification of parameters with a substantial impact on model efficiency. As shown in Fig. 5, the results reveal distinct differences in parameter sensitivity across geological settings—regolith (sandstone), bedrock (quartzite), and bedrock (slate)—highlighting both common and site-specific controls on model behaviour.

Rooting depth (Zr) exhibited a clear influence on model efficiency, particularly in the regolith–sandstone domain. High NSE values were primarily concentrated when Zr was less than 1500 mm, suggesting that shallow effective rooting depth facilitates better simulation of groundwater dynamics. This may reflect the rapid infiltration and recharge processes typical in unconsolidated or weakly cemented layers.

Soil storage coefficient (S) was another consistently sensitive parameter across all settings. In the sandstone region, high model performance was associated with S > 0.06, indicating that the capacity of the soil to retain moisture strongly governs the timing and magnitude of recharge reaching the saturated zone. Similarly, in quartzite and slate terrains, although the influence of S was less pronounced, it still exhibited a positive relationship with NSE.

Upper-layer saturated hydraulic conductivity (K_1_) showed strong sensitivity in the sandstone unit, where better performance was observed when K1 exceeded 6 m/d. This suggests that more conductive upper layers allow for quicker drainage and recharge, supporting timely groundwater level response to rainfall inputs. The relationship in quartzite was more moderate, while in slate terrains, K_1_ appeared less influential, potentially due to the dominance of vertical recharge limitations in fractured bedrock.

In contrast, lower-layer conductivity (K_2_) had weaker influence overall, with only a mild preference for higher values in the sandstone domain. This implies that deep drainage processes play a limited role in short- to mid-term groundwater dynamics under the current model configuration.

Other parameters such as the depletion factor of catchment vegetation (p), baseflow index (BFI), and Weibull shape parameter (k) exhibited negligible or inconsistent influence on model efficiency across all geological units. Their dotty plots were characterized by scattered distributions with no clear trends, suggesting either a low sensitivity or the presence of parameter equifinality. These parameters may be less critical in this study context, where the primary control on groundwater response is governed by shallow subsurface properties and recharge mechanisms.

Overall, the results underscore the importance of unsaturated zone and upper aquifer properties (i.e., Zr, S, and K_1_) in shaping groundwater level dynamics, particularly in more permeable geological settings. These findings not only inform parameter prioritization in future model calibration but also help constrain uncertainty by focusing efforts on the most influential factors.

## Conclusions

This study demonstrates the feasibility of applying a lumped conceptual groundwater model, AquiMod, to simulate groundwater level dynamics in the mountainous region of central Taiwan, where monitoring wells are mostly installed in unconfined or regolith systems and data availability is limited. Long-term trend and correlation analyses reveal that precipitation exerts a dominant control on groundwater variations, with significant decreasing trends observed at the majority of stations, likely reflecting a combination of climatic variability, land-use changes, and local abstraction. The model captures temporal and amplitude fluctuations of groundwater levels reasonably well in most wells, particularly where observation records are complete and local hydrogeological settings are less complex. Model performance is strongly influenced by unsaturated zone parameters such as rooting depth, soil storage, and upper-layer saturated hydraulic conductivity, highlighting their critical role in shaping groundwater responses to rainfall inputs.

Nevertheless, simulation performance deteriorates in some locations with complex geology, pronounced data gaps, or unmonitored anthropogenic influences, emphasizing the limitations of lumped models in representing localized processes. These findings underscore the necessity of incorporating site-specific hydrogeological knowledge and improving observation coverage to enhance model reliability.

Importantly, beyond scientific insights, the results carry practical implications for groundwater management in data-scarce mountainous regions. By identifying dominant controls on groundwater fluctuations and highlighting where simplifications break down, the study provides a framework for prioritizing monitoring efforts, guiding model calibration, and informing adaptive management strategies in similar hydrogeological contexts. The demonstrated utility of a parsimonious model like AquiMod in such settings offers a cost-effective tool for preliminary assessments, resource planning, and policymaking, especially where comprehensive hydrogeological investigations are not feasible. Future efforts should focus on improving model integration with land-use dynamics, groundwater abstraction data, and geological heterogeneity to better support sustainable groundwater resource management. 

## Appendix A. Correlation between precipitation and GWL

**Figure:**
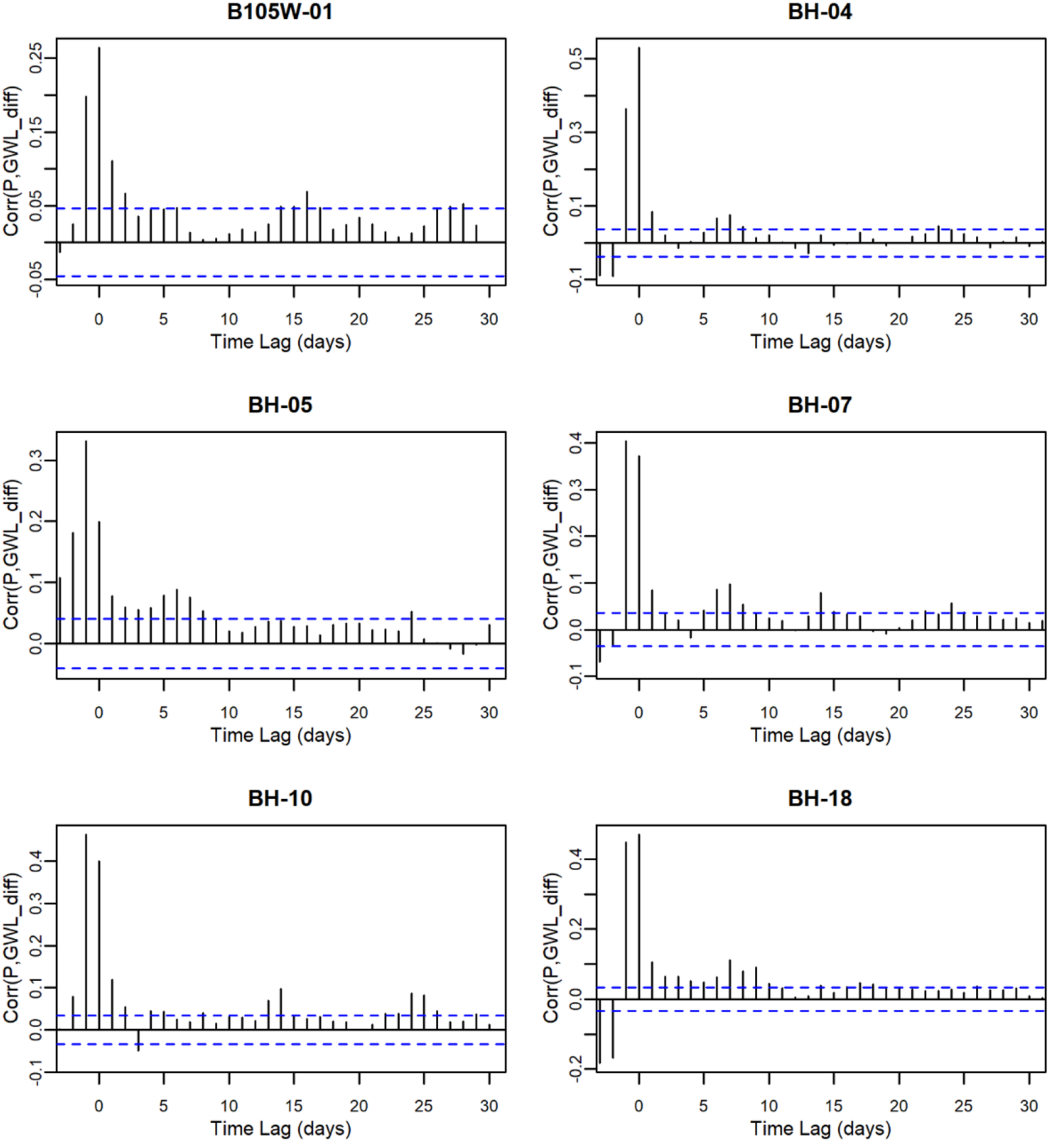

**Figure:**
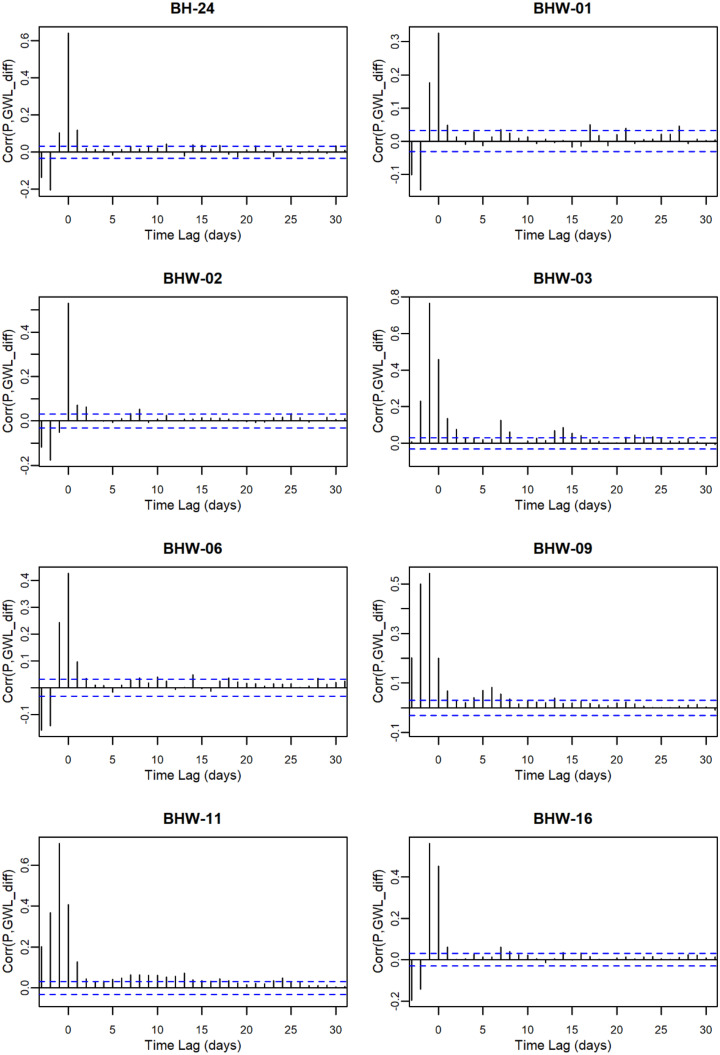

**Figure:**
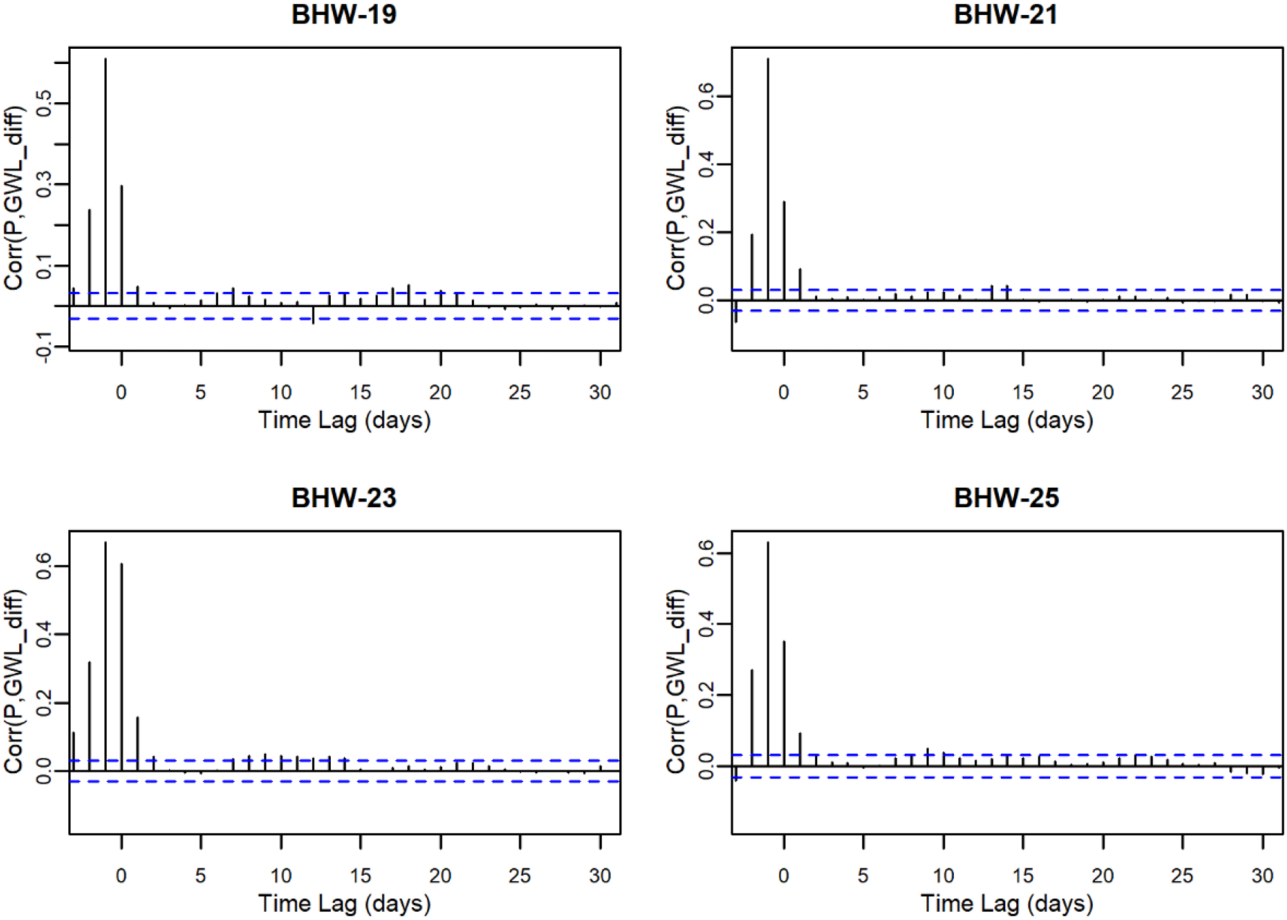

**Figure:**
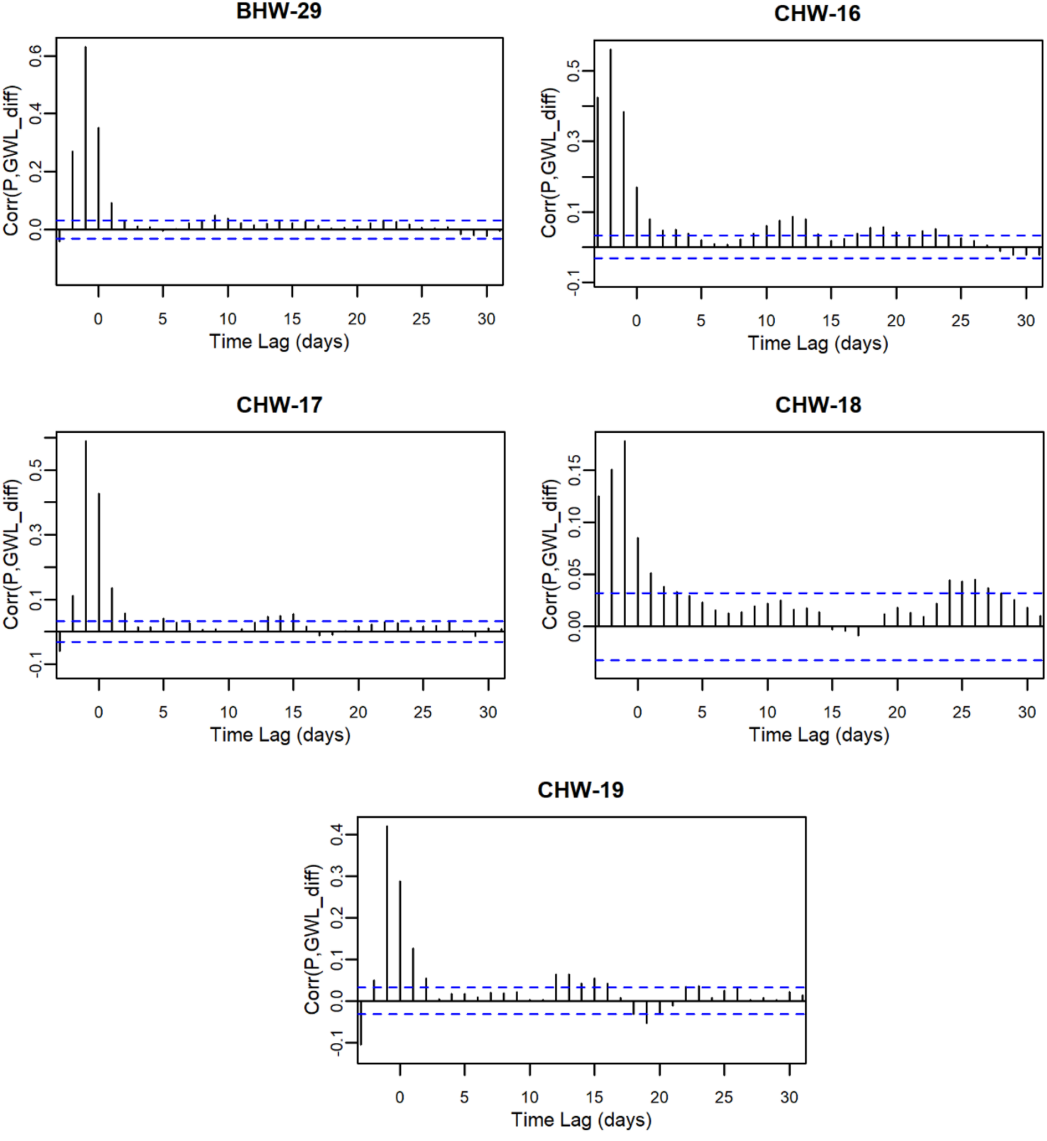

## Data Availability

No datasets were generated or analysed during the current study.
